# A Thermostable Crude Endoglucanase Produced by *Aspergillus fumigatus* in a Novel Solid State Fermentation Process Using Isolated Free Water

**DOI:** 10.1155/2012/196853

**Published:** 2012-07-08

**Authors:** Abdul A. N. Saqib, Ansa Farooq, Maryam Iqbal, Jalees Ul Hassan, Umar Hayat, Shahjahan Baig

**Affiliations:** Food and Biotechnology Research Centre, PCSIR Labs Complex, Ferozepur Road, Lahore 54600, Pakistan

## Abstract

*Aspergillus fumigatus* was grown on chopped wheat straw in a solid state fermentation (SSF) process carried out in constant presence of isolated free water inside the fermentation chamber. The system allowed maintaining a constant vapor pressure inside the fermentor throughout the fermentation process. Crude endoglucanase produced by *A. fumigatus* under such conditions was more thermostable than previously reported enzymes of the same fungal strain which were produced under different conditions and was also more thermostable than a number of other previously reported endoglucanases as well. Various thermostability parameters were calculated for the crude endoglucanase. Half lives (*T*_1/2_) of the enzyme were 6930, 866, and 36 min at 60°C, 70°C, and 80°C, respectively. Enthalpies of activation of denaturation (Δ*H*_D_*) were 254.04, 253.96, and 253.88 K J mole^−1^, at 60°C, 70°C and 80°C, respectively, whereas entropies of activation of denaturation (Δ*S*_D_*) and free energy changes of activation of denaturation (Δ*G*_D_*) were 406.45, 401.01, and 406.07 J mole^−1^ K^−1^ and 118.69, 116.41, and 110.53 K J mole^−1^ at 60°C, 70°C and 80°C, respectively.

## 1. Introduction

Endoglucanases (EC 3.2.1.4) constitute a large proportion of the group of enzymes collectively known as cellulases which are the 3rd largest enzymes sold worldwide and have applications in a number of industries [1]. Their demand is increasing fast especially because of the emergence of second-generation-advanced biofuel industries which require tremendous amounts of various enzymes in their processes [2, 3]. In order to decrease process costs and increase the efficiencies, it is desirable to use thermostable enzymes in the industrial processes [3]. However, most cellulases are not stable at high temperatures [4], and a number of efforts are being made in order to obtain thermostable cellulases [3].

Solid state fermentation (SSF) has long been used for the production of cellulases and other enzymes or bioproducts [5]. It was recently shown that *A. fumigatus *produced a more thermostable endoglucanase using SSF than that produced through a submerged process [6]. SSF is carried out in the absence or nearly absence of free water in the fermentation medium [5, 7]. In many of the reported experiments, moisture level of the substrate is neither monitored nor controlled after the onset of the SSF process. Even when monitored, it is often estimated “off-line” thus creating technical problems regarding determining the actual water activity (*a*_*w*_) of the substrate medium [8]. The problems can be overcome by designing a system which would allow keeping the water activity of the medium constant during an SSF process [9].

Significant amount of heat is produced by the microbial activity during the course of SSF which can substantially change the water activity of the substrate. For example, temperature can rise up to 70°C during composting in heaps [7] which may result in significant amount of water to be lost through vaporisation. Therefore, additional supplementation of water is deemed advantageous during the course of large-scale SSF processes [7]. However, it may not be desirable to increase the water activity of a solid substrate beyond certain point, even for a short period of time, because a high moisture level in the SSF may result in decreased substrate porosity thus preventing the oxygen penetration and also helping bacterial contamination to occur [9]. 

There have been a few reports on designing an SSF system in which water activity may be kept constant during the course of fermentation. Gervais and Bazelin [10], for example, proposed an SSF system comprising multiple chambers which allowed humid air with set moisture level to circulate through the fermentation chamber. Some other attempts have also been made over the past years to address the issue of controlling water activity during SSF [11].

This study reports thermostability of a crude endoglucanase produced by *A. fumigatus *using a modified SSF approach which featured constant presence of isolated liquid water inside the fermentation chamber without its direct contact with the substrate. Thermostability of the enzyme preparation is also compared with other reported enzymes and is discussed in detail.

## 2. Materials and Methods

All chemicals were purchased from Sigma-Aldrich, St. Louis, MO, USA, unless otherwise mentioned.

### 2.1. Fungal Strain

A previously isolated fungal strain (SMN1) which was identified by the First Fungal Culture Bank of Pakistan, Institute of Mycology and Plant Pathology (IMPP), University of the Punjab, Lahore, Pakistan, as an *Aspergillus fumigatus* sp. (IMPP Reference: 922) was used for enzyme production during these experiments. The fungus was maintained on Vogel's minimal medium (VM) agar overlaid with a Whatman no. 1 filter paper (FP) disc as described previously [6].

### 2.2. Fermentation Experiments

**Figure 1:**
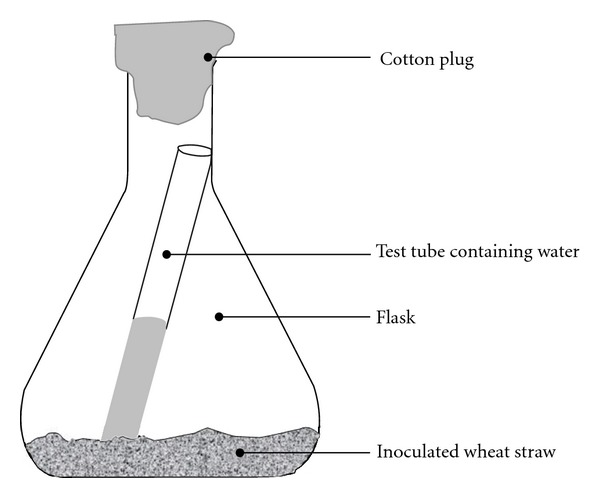
Prototype fermentor: the Erlenmeyer flask contained solid substrate (wheat straw) and inoculum along with a test tube half filled with water. Continuous presence of liquid water during the course of fermentation process ensured a constant vapour pressure inside the fermentation chamber.

Two g chopped wheat straw (5–10 mm length) along with 5 mL Vogel's medium [12] was put into 100 mL Erlenmeyer flasks. The flasks were then vigorously shaken so that the added liquid is evenly distributed throughout the substrate. Then, a test tube half filled with distilled water was placed inside the flasks (Figure 1) in order to ensure a constant supply of water vapours inside the fermentation chamber during the course of fermentation. The flasks were then tightly plugged with cotton and autoclaved at 121°C and 15 psi for 30 minutes. Inoculation of the substrate, incubation of the inoculated substrate at 30°C for one week, and subsequent enzyme extraction using 0.05 M acetate buffer pH 4.8 were performed as described previously [6]. The spore suspensions used as inocula in these experiments contained approx. 10^6^ spores each.

### 2.3. Protein Estimation

Total protein in the crude enzyme extract was measured according to Lowry et al. [13]. Five mL alkaline copper reagent was added into 0.5 mL of enzyme sample in glass test tubes in triplicate. A control blank was prepared using water instead of enzyme solution. The mixtures were kept at room temperature (25°C) for 10 minutes, followed by the addition of 0.5 mL of Folin reagent into each of the test tubes. The test tubes were then kept at room temperature for another 30 min, after which absorbance was measured at 660 nm and translated into protein concentration using a standard curve made by using casein as standard.

### 2.4. Enzyme Assay

Crude endoglucanase (CMCase) activity was measured using carboxymethyl cellulose as described previously [14].

### 2.5. Characterization of the Optimum Temperature

Temperature of maximum enzyme activity (optimum temperature) was estimated by performing the CMCase assay at various temperatures ranging from 25°C to 80°C and drawing an Arrhenius plot of the data as described by Siddiqui et al. [15].

### 2.6. Thermostability Analysis

Thermostability of the crude endoglucanase preparation was evaluated by incubating enzyme samples, in the absence of substrate (CMC), at 50°C, 60°C, 70°C, and 80°C for various lengths of time ranging from 0 to 120 minutes. The data were plotted and analyzed as described previously [6]. Half lives (*T*_1/2_) of the enzyme at various temperatures were calculated using (1):

(1) $$\begin{array}{c} T_{1/2}=\ln\frac{2}{k_{d}}=\frac{0.693}{k_{d}}. \end{array}$$

Other thermostability parameters, such as, enthalpy of activation of the thermal denaturation (Δ*H*_D_*), entropy of activation of thermal denaturation (Δ*S*_D_*), and the Gibbs-free energy of activation of thermal denaturation (Δ*G*_D_*) were calculated using following equations:

(2) $$\begin{array}{c} \Delta H_{D}^{*}=E_{a(D)}-RT\\ \Delta G_{D}^{*}=-RT\times \ln\left(\frac{k_{d}\times h}{k_{B}\times T}\right),\\ \Delta S_{D}^{*}=\frac{\left(\Delta H_{D}^{*}-\Delta G_{D}^{*}\right)}{T}, \end{array}$$

where, *E*_*a*(*D*)_ is activation energy for the thermal denaturation of the enzyme; *R* is universal gas constant = 8.314 J K^−1^ mol^−1^; *T* is absolute temperature (Kelvin); *k*_*d*_ is first order rate of thermal inactivation of the enzyme activity; *h* is planck's constant = 6.63 × 10^−34^ J s; *k*_*B*_ is boltzmann constant = 1.38 × 10^−23^ J K^−1^.

### 2.7. Statistical Analysis

Student's *t*-test and other statistics were applied where required using GraphPad InStat software.

## 3. Results

**Figure 2:**
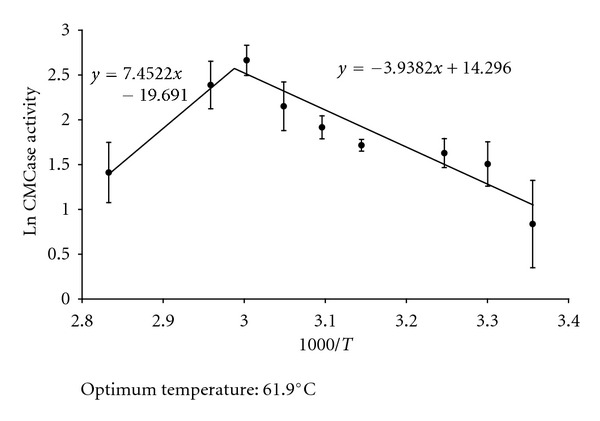
First-order Arrhenius plot showing the effect of temperature on activity of crude endoglucanase produced by *A. fumigatus* grown for 7 days under the SSF_H_2_O_ conditions using wheat straw as the carbon source.

**Figure 3:**
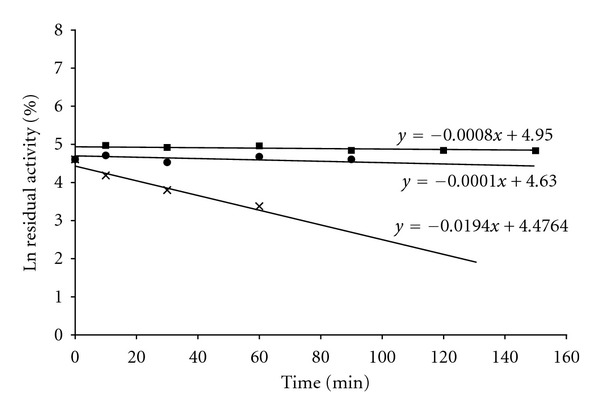
First-order plot for the effect of temperature on enzyme activity of crude endoglucanase produced by *A. fumigatus* after 7 days of growth under SSF_H_2_O_ conditions using wheat straw as the solid substrate. The enzyme samples were incubated at 60°C (●), 70°C (■), and 80°C (x) for various lengths of time and then assayed for the residual activity.

**Figure 4:**
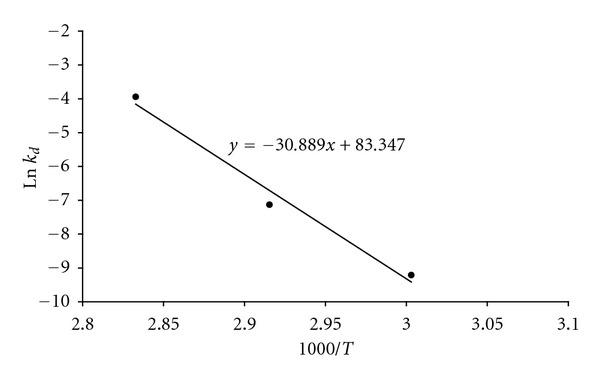
First-order Arrhenius plot for determination of activation energy of denaturation (*E*_*a*(*D*)_) of crude endoglucanase from the *A. fumigatus* grown under the SSF_H_2_O_ conditions. Note: Values of first-order rate constants (*k*_*d*_) for thermal denaturation of the enzyme at different temperatures were obtained from the slopes in Figure 3.

The crude endoglucanase (SSF_H_2_O_-EG) produced by *A. fumigatus *through the modified SSF technique described in this paper, that is, SSF carried out in constant presence of free liquid water inside the fermentation chamber (SSF_H_2_O_) (Figure 1), showed maximum activity for substrate (CMC) hydrolysis at 61.9°C (Figure 2). It was higher than many of the previously reported endoglucanases, such as, those reported by Thongekkaew et al. [16], Siddiqui et al. [15], and a number of other examples quoted by De Vries and Visser [17]. In addition, the SSF_H_2_O_-EG possessed a very long half life (*T*_1/2_) as well which was 6930 min or 116 hrs at 60°C (Figure 3 and Table 2), in other words it would take about 5 days for the enzyme activity of SSF_H_2_O_-EG to drop down to one half of its original activity while working close to its temperature of maximum activity, 61.9°C. In addition to a longer *T*_1/2_, the SSF_H_2_O_-EG also had a higher melting temperature (*T*_*m*_), 88°C (Figure 4) which is an indication of the tendency of an enzyme to keep its 3D structure intact and functional at the given temperature. Temperature coefficients (*Q*_10_) of SSF_H_2_O_-EG were between 1.4 and 1.5 at 40–60°C (Table 1).

**Figure 5:**
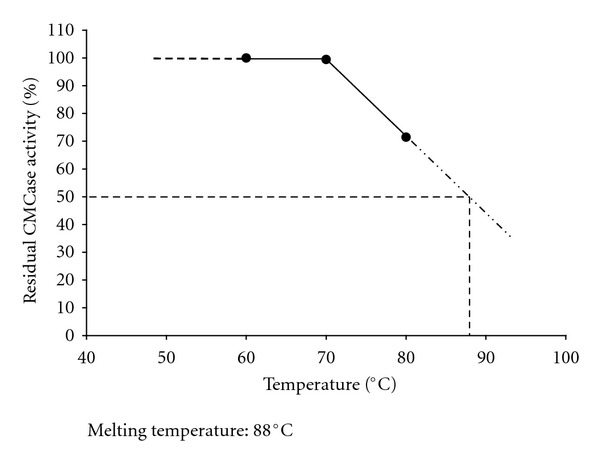
Determination of melting temperature (*T*_*m*_) for the crude endoglucanase produced by *A. fumigatus* grown under SSF_H_2_O_ conditions. The *T*_*m*_ corresponds to the temperature at which the enzyme activity drops down to the 50% of the initial activity.

Activation energy of denaturation (*E*_*a*(*D*)_) for the SSF_H_2_O_-EG was 256.811 K J mole^−1^ (Figure 5). Enthalpy of activation of denaturation (Δ*H*_D_*) for SSF_H_2_O_-EG was just over 250 k J mole^−1^ at various studied temperatures (Table 2). Entropy of activation of denaturation (Δ*S*_D_*) was over 400 J mole^−1^ K^−1^ at all temperatures, whereas the Gibbs-free energy of activation of denaturation (Δ*G*_D_*) values for SSF_H_2_O_-EG ranged between 119 and 111 k J mole^−1^ at temperatures ranging between 60 to 80°C (Table 2).

## 4. Discussion

The crude endoglucanase refers to the overall activity of the enzyme preparation which may contain more than one enzyme. It is considered advantageous to use crude enzymes in many bioprocesses, such as, those used in biofuel industries, in order to reduce the overall process cost. A crude enzyme preparation may also contain additional activities which may act as auxiliary activities, thus, improving the enzymatic hydrolysis [6]. 

Enzymes having high thermostabilities and high temperatures of optimum activities are sought after for industrial uses where processes often run at high temperatures, typically above 50°C [3, 18]. The SSF_H_2_O_-EG reported in this study had a relatively very high temperature of maximum activity; however, it may be noteworthy that a high temperature of maximum enzyme activity on its own may not be a useful enough feature, particularly if the process has to run for a long period of time, because the enzymes may die out quickly. In order to be useful enough it must withstand elevated temperature for a longer time period, maintaining most of its activity for at least the duration of the process. Therefore, a more reliable parameter of enzyme activity, half life (*T*_1/2_), must be taken into account in conjunction with the temperature of maximum activity.

The *T*_1/2_ of SSF_H_2_O_-EG was remarkably longer at 60°C than any of the previously reported endoglucanases to be best of our knowledge. For example, the one from *A. oryzae *had a *T*_1/2_ of only 21 min, 8 min and 2 min at 50°C, 53°C, and 56°C, respectively, note the low temperature range applied-[19], a crude endoglucanase of *A. niger *had a *T*_1/2_ of only 43 min at 50°C [20], another crude endoglucanase preparation from *A. fumigatus* obtained through conventional SSF process had *T*_1/2_ of 248 min at 60°C [6] and an endoglucanase from *A. niger* had *T*_1/2_ of 167, 88, 66, and 69 min at 50°C, 55°C, 60°C, and 65°C, respectively, [21]. The *T*_1/2_ of SSF_H_2_O_-EG reported herein was quite long even at higher temperatures as well, that is, 866 min and 36 min at 70°C and 80°C, respectively (Table 2). A long half life suggests that the enzyme was thermostable and should also had a high melting temperature (*T*_*m*_) as well. The *T*_*m*_ is an intrinsic property of proteins which corresponds to the change in proteins' secondary and tertiary structures upon heating which leads to distortion of its active site(s) and a consequent loss of activity [22]. Therefore, a high *T*_*m*_ of SSF_H_2_O_-EG (Figure 4) was a good indication that the enzyme could withstand a higher temperature without losing its functional 3D structure and activity. This observation was backed by the temperature coefficients (*Q*_10_) values for SSF_H_2_O_-EG. The *Q*_10_ is a factor by which the rate of enzyme reaction changes for every 10 degree rise in temperature [6], and relatively low values for SSF_H_2_O_-EG showed that a change in temperature would not have significant effect on the tertiary protein structure of the enzyme at up to 60°C.

Enzymes with a high activation energy of denaturation (*E*_*a*(*D*)_) are more resistant to thermal denaturation than those having lower *E*_*a*(*D*)_ [6]. The *E*_*a*(*D*)_ for the SSF_H_2_O_-EG was far higher than the *E*_*a*(*D*)_ of the endoglucanase produced by the same fungal strain under ordinary SSF process conditions (154.7 K J mole^−1^) [6]. It was also higher than a number of other reported endoglucanases, such as, a purified endoglucanase from *A. niger *with *E*_*a*(*D*)_ of 40 K  J mole^−1^[21]. However, an endoglucanase of *A. oryzae *has been shown to have even higher *E*_*a*(*D*)_, which was 378 K J mole^−1^[19].

Enthalpy of activation of denaturation (Δ*H*_D_*) for SSF_H_2_O_-EG, which is the total amount of energy needed for activation of the denaturation process of the enzyme,was significantly higher than Δ*H*_D_* for the previously reported SSF-EG (~152 K J mole^−1^) [6]. Other workers have reported values lower as well as higher than this. For examples, a value of 37 K J mole^−1^ at various temperatures ranging from 45 to 65°C has been reported for an endoglucanase from *A. niger *[21] and 375 K J mole^−1^ between 44 to 56°C for another endoglucanase from *A. oryzae* [19]. It may be noteworthy that calculation of Δ*H*_D_* is based on *E*_*a*(*D*)_ values and, therefore, the former tends to follow the same trend that in later.

Gibbs-free energy of activation of denaturation, Δ*G*_D_*, which determines whether a reaction would occur or not, is an important thermostability parameter. A smaller or negative Δ*G*_D_* implies a favourable reaction, that is, thermal denaturation of protein in this context. The higher the Δ*G*_D_*, the more resistant the protein/enzyme is towards thermal denaturation [6]. Values of Δ*G*_D_* for SSF_H_2_O_-EG (Table 2) depicted a high thermostability. The Δ*G*_D_* value for SSF_H_2_O_-EG was higher than those of the previously reported enzyme of the same fungal strain (SSF-EG) at various temperatures [6]. It was also higher than another endoglucanase from *A. niger* [21] and the one from *A. oryzae* [19].

## 5. Conclusions

A number of thermodynamics parameters used in this study indicated that SSF_H_2_O_-EG was a thermostable enzyme preparation. A high Thermostability of SSF_H_2_O_-EG could possibly be the result of keeping air moist throughout the course of fermentation. Moisture level plays an important role in SSF, and the spectrum of fungal secondary metabolites is known to differ with changes in moisture level of the surrounding medium which has been attributed to switching on and off of certain genes. Changes in water activity of the medium could, therefore, be exploited in order to obtain desirable bioproducts [9]. Above results showed that a highly thermostable crude endoglucanase was produced by a fungal strain when isolated free water was introduced into the conventional SSF system, thus, creating a constant vapor pressure at the given temperature which would allow moisture content of the substrate to remain constant throughout the fermentation period. This study opens up new opportunities to use this fermentor designs, for example, to study metabolic changes in *A. fumigatus* as well as other microorganisms too. It also leads to the possible research on combining metabolomics and genomics approaches in order to identify the transcriptional and translational changes in response to changes in surrounding moisture level.

## Figures and Tables

**Table 1:** Activity profile of culture filtrates of *A. fumigatus* grown for one week under SSF_H_2_O_ conditions. The fermentation was carried out at 30^°^C for 7 days under static conditions with manual shaking once a day.

Protein concentration	87 (±5.9) (*μ*g/mL)
CMCase activity^∗^	868 (IU/mL)
Specific activity	9977 (IU/mg)
Activation energy^∗∗^ (*E*_*a*_)	32.7 K J mole^−1^
Temperature coefficients (*Q*_10_)	1.50 at 40^°^C
	1.46 at 50^°^C
	1.43 at 60^°^C

*Enzyme activity was measured at 60^°^C, that is, near to the optimum temperature of the enzymes, **E_*a*_ was calculated based on Figure 2.

**Table 2:** Kinetic and thermodynamic parameters of irreversible thermal denaturation of crude endoglucanase from *A. fumigatus* grown for 7 days under SSF_H_2_O_ conditions.

Temperature		*k*_*d*_^$^ (min^−1^)	T_1/2_ (min)	Δ*H*_D_* (K J mol^−1^)	Δ*G*_D_* (K J mol^−1^)	Δ*S*_D_* (J mol^−1^ K^−1^)
^ °^C	K					
60	333	0.0001	6930	254.04	118.69	406.45
70	343	0.0008	866	253.96	116.41	401.01
80	353	0.0194	36	253.88	110.53	406.07

^$^ **: **Values of *k*_*d*_ were obtained from Figure 3.

Note: Activation energy of denaturation (*E*_*a*(*D*)_) used to estimate Δ*H*_D_* was calculated using equation: *E*_*a*(*D*)_ = −(slope × *R*) = 256.811 K J mole^−1^ where the value of slope was obtained from Figure 4.
